# The effectiveness of Tuina in managing chronic non-specific low back pain: A protocol of a multicenter international randomized controlled trial

**DOI:** 10.1097/MD.0000000000028883

**Published:** 2022-02-18

**Authors:** Xuan Zhou, Juan Yang, Qing-yu Ma, Yu Guo, Ke-jie He, Long-bin Shen, Qiao Fan, Cheong Kwok Chee Philip, Tay Boon Keng, Tan Ia Choo Celia, Brent A. Bauer, Jia-xu Chen

**Affiliations:** a Formula-Pattern Research Center, School of Traditional Chinese Medicine, Jinan University, Guangzhou, Guangdong Province, China; b Division of General Internal Medicine, Mayo Clinic, Rochester, MN; c Department of Acupuncture, First Affiliated Hospital of Jinan University, Guangzhou, Guangdong Province, China; d Rehabilitation Medicine Center, First Affiliated Hospital of Jinan University, Guangzhou, Guangdong Province, China; e Center for Quantitative Medicine, Duke-NUS Medical School, Singapore; f Department of Physiotherapy, Singapore General Hospital, Singapore; g Department of Orthopedic Surgery, Singapore General Hospital, Singapore.

**Keywords:** chronic non-specific low back pain, clinical trials, complementary medicine, rehabilitation medicine, traditional Chinese medicine, Tuina

## Abstract

**Background:**

Chronic non-specific low back pain (CNLBP) is a common complaint about medical care and carries a heavy social burden. The efficacy of Tuina (TN) or physiotherapy (PT) for CNLBP has been evaluated in previous systematic reviews. However, there is no high-quality evidence to support the efficacy of Tuina. Therefore, this study aims to conduct a large-scale, multicenter, high-quality clinical trial to provide evidence for Tuina to treat CNLBP.

**Methods:**

This is a multicenter, assessor-, and analyst-blinded, randomized controlled trial with 3 parallel arms: TN, PT, and TN combined with PT (Tuina combined with physiotherapy) group. Six hundred twelve eligible CNLBP patients will be randomly assigned to the groups in a 1:1:1 ratio in 3 centers. The TN intervention includes 9-step routine techniques, while the PT intervention includes a physiotherapy treatment plan based on a patient's symptoms. The interventions for both groups will last for 30 minutes and will be carried out for 6 sessions in 8 weeks. The primary outcome will be the visual analog scale pain score. And the secondary outcomes will include the Oswestry Disability Index, spinal range of motion, 36-item short-form health survey. Safety evaluation will be recorded during the whole study. All data in this randomized controlled trial will be analyzed by SAS 9.4.

**Discussion:**

The results of this trial will provide evidence to evaluate the efficacy of Tuina's value as a treatment for CNLBP.

**Trial registration:**

Chinese Clinical Trial Registry (ChiCTR2000040288, November 27, 2020).

## 1 Introduction

Low back pain (LBP) is a common clinical symptom prompting patients to seek medical care. 80% of adults experience LBP during their lifetime.^[1]^ It is the leading cause of disability worldwide, with a global prevalence of 7.3% in 2016.^[2]^ Usually, most people recover with or without treatment, however, a relapse of the pain is common and 10% to 15% of LBP patients may develop chronic low back pain (CLBP).^[3]^ CLBP can be categorized into sciatica/nerve root pain syndrome, specific low back pain, and non-specific low back pain.^[^4^,^^5^^]^ The common causes of CLBP can include spinal injury, lumbar muscle strain, degenerative spinal intervertebral disc and vertebra joints, and kidney disease in women. 90% of CLBP patients do not have a clearly identifiable cause of pain, classified as chronic non-specific low back pain (CNLBP).^[^6^,^^7^^]^ CNLBP refers to pain and discomfort localized in the area between the costal margin and the inferior gluteal fold, not resulting from an identifiable or known specific pathology, with or without referred leg pain of >12 weeks’ duration.^[^8^,^^9^^]^ CNLBP's symptom onset, recovery, and clinical outcomes are influenced by biological, psychological, and social factors; therefore, its management is often complicated and multimodal.^[10]^

As the pathoanatomical cause of CNLBP remains an enigma, no specific evidence-based clinical practice method has been recommended for medical providers to manage CNLBP.^[^11^,^^12^^]^ According to the Consensus on the management of LBP,^[13]^ there is a wide variety of approaches recommended for CNLPB, such as patient education, self-management, physiotherapy, psychological therapy, medication, injection, ablation techniques, or surgery. Although these treatments have shown some efficacy, there is no conclusive evidence for their continued use in the mainstream medicine or sufficient knowledge of the extent of their side effects.^[12]^ Nonetheless, a multidisciplinary eclectic rehabilitation strategy is often recommended for patients with CNLBP.^[14]^ More and more people have turned their attention to complementary and alternative medicine (CAM) treatment.^[^15^,^^16^^]^ At present, Tuina therapy has been widely accepted as a CAM modality.^[17]^

Tuina (TN), also named “Tui Na,” literally means “pushing (and) grasping” and has been used in China for thousands of years. It is a non-pharmacological manual therapy in traditional Chinese medicine (TCM), which is mainly applied to the meridians or acupoints, which are pathways for the qi and blood of the human body, by manipulation of pushing, grasping, pressing, rubbing, etc. Over the years, TN has developed currently into one of the most popular TCM therapies with a wide range of techniques for various body locations.^[18]^ It is proposed to treat diseases by removing the obstruction in Meridian collaterals and relieving pain by reducing inflammation, separating tendon adhesions, improving circulation, and re-aligning minor joint disorders.^[19]^ The use of TN as a treatment intervention has also been studied for other forms of pain management, pediatric cerebral palsy disorders, neurological and musculoskeletal conditions, and rehabilitation.^[20]^ In recent years, many studies have emerged to evaluate the effectiveness of TN to manage LBP.^[^21^–^23^]^ A systematic review and meta-analysis^[23]^ assessed the efficacy of TN-focused integrative Chinese medical therapies on LBP inpatients, incorporating 20 randomized controlled trials (RCTs). This review found that TN might be practical (SMD: 1.17; 95% CI 0.75–1.60 on pain; SMD: 1.31; 95% CI 0.49–2.14 on functional status) for inpatients with LBP, although more high-quality RCTs with longer follow-up were required to raise the level of evidence for its use for LBP.

There are also many studies on the therapeutic effect of TN on patients with CNLBP.^[^21^,^^24^^–^28^]^ Still, most of these trials involve the combination of TN with other CAM therapies^[^21^,^^24^^,^^25^^,^^27^^]^ or were conducted on a small sample size with poor methodological quality. There is insufficient evidence for the effective treatment of CNLBP using TN. TN is still not included in guidelines for LBP treatment in most countries,^[29]^ especially those using mainly Western forms of medicine. Therefore, the purpose of the study is to evaluate the effectiveness and safety of TN in the management of CNLBP.

## 2 Methods and analysis

### 2.1 Study design

This is a prospective, multicenter, assessor-, and analyst-blinded RCT of TN for CNLBP. Six hundred twelve patients will be allocated to one of the 3 groups: TN, PT, or Tuina combined with physiotherapy (T&P).

**Figure 1:**
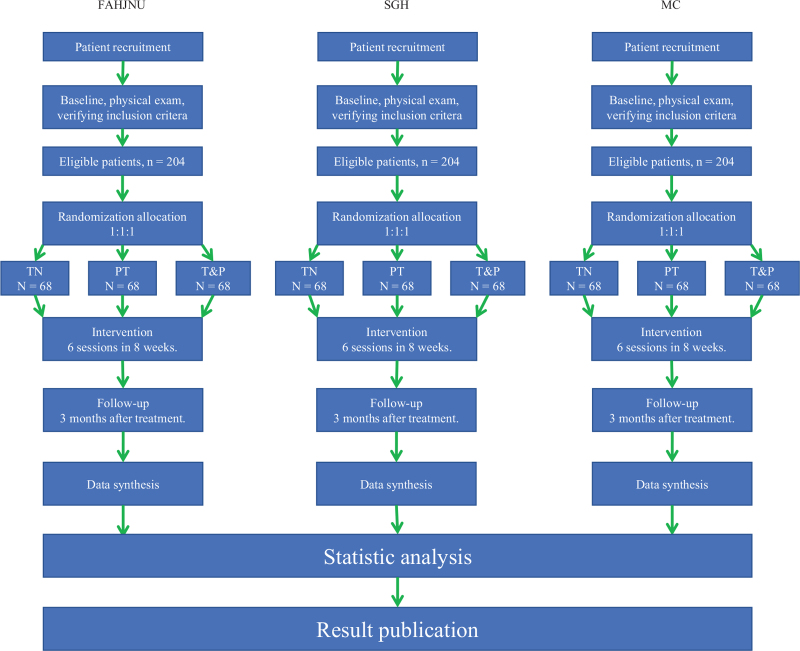
Flow chart of the study. A total of 612 participants will be randomized to the 3 groups. The study period will consist of the baseline, end of 2-month intervention, 3 months post-intervention. FAHJNU = First Affiliated Hospital of Jinan University, MC = Mayo Clinic, SGH = Singapore General Hospital.

**Figure 2:**
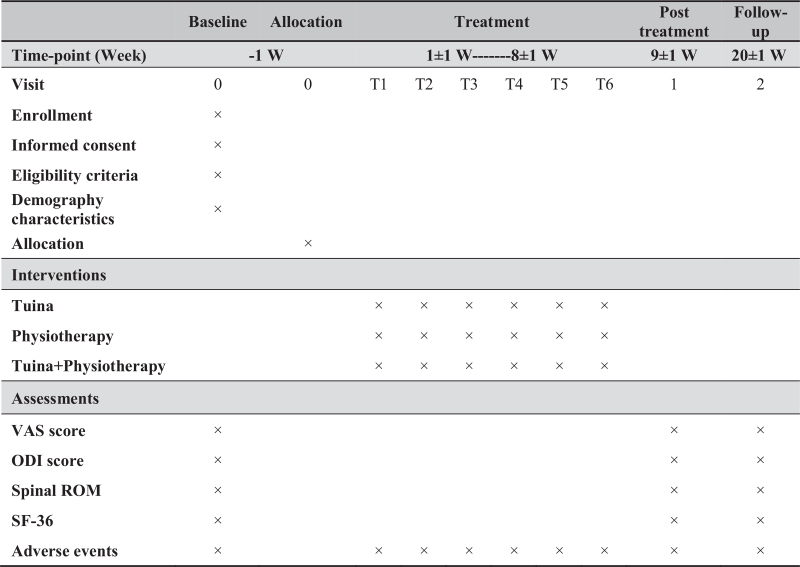
Schedule of the study. Each session lasts about 30 minutes, for 6 sessions, over a total of 8 weeks. ODI = Oswestry Disability Index, RCT = randomized controlled trial, ROM = range of motion will be recorded at these points of assessment, SF-36 = 36-item Short-Form Health Survey, VAS = visual analog.

The study design is illustrated in the flow chart in Fig. 1 and the study schedule is presented in Fig. 2. And this protocol follows the 2013 SPIRIT^[30]^ (Standard Protocol Items: Recommendations for Interventional Trials) Guideline.

### 2.2 Ethics and registration

The study protocol has been approved by the Ethics Committee /Institutional Review Board of the First Affiliated Hospital of Jinan University (KY-2019–042), the Mayo Clinic (20–003549), and the Singapore General Hospital (CIRB 2020/2798). And the approved protocol version is 11.0, at October 2020. Any protocol amendments will be detailed in the trial registration. We will follow the International Conference Guideline for Good Clinical Practice to ensure that the data and the results are credible. Before starting the study, investigators will inform patients of relevant information, including this clinical trial's potential benefits and possible harms. All the information provided from patients, such as personal health information, would be kept confidential.

This trial is recruiting patients now. Participant recruitment started in December 2020 and is expected to end in December 2022. It is registered on November 27, 2020 with the Chinese Clinical Trial Registry, and the registration number is ChiCTR2000040288.

### 2.3 Participants and recruitment

Eligible participants include patients diagnosed with CNLBP according to the Chinese Association for the Study of Pain (CASP) Consensus for the management of CNLBP (Inclusion 1, 2, 3).^[13]^ Patients presenting for the first time to either the Rehabilitation (Physiotherapy), General Internal Medicine Department, or Acupuncture Department in the hospital or clinic for low back pain for over 3 months, will be informed of this trial. If the patient expresses interest, a clinical trial communicator/research assistant will contact them to briefly introduce the trial and assess their suitability based on the study's inclusion and exclusion criteria.

### 2.4 Inclusion criteria

Those who meet the following criteria are eligible to participate in the study:

(1) Pain and discomfort of unknown etiology between the costal margins and inferior gluteal folds with or without referred pain to the lower limbs, lasting for >12 weeks.
(2) Has at least one symptom of spinal muscle weakness, stiffness, limited mobility, or reduced spinal coordination. The symptoms of pain are lessened or disappeared after bed rest, while pain symptoms are aggravated after bending over, sitting for a long time, or standing for a long time.
(3) Physical examination showed increased muscle tension or a significant localized tenderness point (trigger point) in the painful area, with a negative straight-leg raising test and no signs of nerve root lesions.
(4) Patients are older than 21 and younger than 75 years of both sexes.

### 2.5 Exclusion criteria

Those who meet any of the below exclusion criteria shall be excluded from the study:

(1) Patients with LBP associated with nerve root dysfunction.
(2) Patients with severe skin diseases (e.g., skin cancer, erysipelas, severe eczema, severe dermatitis, severe psoriasis, and severe hives lupus).
(3) Serious spinal pathologies such as fractures, tumors, inflammatory, and infectious diseases.
(4) History of spine surgery.
(5) Serious cardiovascular or metabolic disorders, such as heart failure, severe osteoporosis.
(6) Patients diagnosed with cognitive issues such as major depression, and moderate to severe dementia, and/or severe psychiatric diseases (such as schizophrenia, bipolar affective disorder, paranoid psychosis).
(7) Women who are pregnant or of childbearing potential and are likely to become pregnant during the treatment phase but are not willing to use a reliable form of contraception will also be excluded. Reliable forms of contraception include oral contraception, diaphragm or condom (with spermicide), injections, intrauterine devices, surgical sterilization, and abstinence.

### 2.6 Suspension criteria

Subjects may discontinue from the study after discussions with the clinicians for the following reasons:

(1) The subject has an adverse reaction to the intervention.
(2) The subject's pain score decreased to 0.
(3) The subject wishes to withdraw from the clinical study.
(4) The subject wishes to attend other interventions, which may result in the study bias, such as attending chiropractic or other massage therapy or non-traditional spinal treatment.

Those who meet any of the above suspension criteria shall be suspended. However, all subjects’ responses will be recorded to determine the reasons for suspension from the study and the intention-to-treat analysis.

### 2.7 Sample size calculation

This is a randomized controlled trial with 3 parallel arms. The test groups are the TN and T&P groups; the control group is the PT group. The visual analogue scale (VAS) score is the primary outcome. We use ANOVA to calculate the sample size. According to the previous report,^[24]^ the mean (standard deviation) difference of the VAS score of pre- and post-treatment in the TN group, T&P group, and PT group is 1.4 (0.8), 1.9 (0.9), and 1.65 (0.85), respectively. Assuming *α* = 0.05 (2-sided), *β* = 0.80, 171 subjects (57 subjects per group) are needed to achieve an 80% probability of detecting difference (PASS software, Ver. 11, NCSS, LLC. Kaysville, Utah; www.ncss.com). When considering a potential dropout risk of 15%, a total of 204 subjects (68 subjects per group) will be required as a sample size for statistically significant results at each center.

Stratified by 3 centers, the combined sample size is 612 for a meta-analysis.

### 2.8 Randomization

In this study, we will use a blocked randomization method. Each site will run the randomization independently, and the same randomization method will be adopted. A statistician will use SAS 9.4 (SAS Institute Inc., Cary, NC) with a “proc plan” program to produce the randomizing scheme. After production, the scheme will be signed and sealed by the staff who produces it and kept by other staff participating in this trial. It will not be allowed to be checked by anyone except the Principal Investigator. Physicians in each center will be responsible for getting random numbers.

### 2.9 Blinding

Due to the inability to blind patients, the practitioners, physiotherapists, and patients are not blinded.

The assessment of clinical efficacy will be performed by a clinical assessor who will be masked to the treatment assignment. The clinical researcher, assessor, and statistician do not share study information during this clinical trial.

### 2.10 Interventions

Participants will receive 6 sessions of treatment over 8 weeks (every 1–2 weeks per session). Each session will last for 30 minutes. Trained and experienced TCM practitioners or physiotherapists who have studied massage and must have received professional training and passed a test to ensure consistency of study methods before participating in the trial will provide the treatment. (Appendix 1).

### 2.11 Outcome measurements

The primary outcome measure assesses the efficacy of massage therapy for the treatment of CNLBP: change in low back pain measured by the 100 mm VAS^[31]^ over the 2-month treatment. Secondary outcome measures included the Oswestry Disability Index^[32]^ (ODI), spinal range of motion^[33]^ (ROM), and the 36-item short-form health survey^[34]^ (SF-36). Fig. 2 demonstrates all measurements and measuring time points.

### 2.12 Safety evaluation

Since TN and PT are both non-invasive external treatments, the side effects are minor, but in the course of operation, if the technique is misused, there may be accidents such as muscle, bone, or joint damage of the spine or other parts of the body. Therefore, the response to an adverse event should strictly follow the standard operating requirements to prevent accidents.

Pre- and post-treatment of each treatment session, the researchers will review the patient's symptoms according to the following safety classification:

Level 1: Safe, without any adverse reactions and treatment can continue.
Level 2: Relatively safe, with minor adverse reactions, treatment can be continued without any additional remedial treatment for the injury.
Level 3: There are safety problems, moderate adverse reactions, and treatment can continue after treatment of the injury.
Level 4: Suspension of participation in research due to adverse reactions.
Adverse events such as changes in pain, syncope, vertigo, and lumbar function degradation, will be carefully recorded in the case report form.

### 2.13 Follow-up

To evaluate the interventions’ short-term efficacy and safety, we will follow up with participants for 3 months after the trial. In the 20th week, the outcome assessor will telephone participants to investigate their low back pain recurrence. Patients can also inform the assessors of their clinical symptoms and AEs face to face or by email, text message, or WeChat.

### 2.14 Data collecting and monitoring

All baseline assessment, end of 2-month intervention, 3 months post-intervention data will be collected by the CRC blind to the treatment modality or treatment groups. All subjects’ adverse events and additional treatment during the follow-up period will also be recorded.

This trial uses a paper-based case report form (CRF) and an electronic data management system. All data will be recorded by CRC in CRFs. Completed CRFs are reviewed by Clinical Research Associate (CRA), from a third-party company, independent of the research team and blinded to group allocation. All data administrators have data analysis qualifications and are trained uniformly. To ensure the accuracy of the data, 2 data administrators independently enter the information and proofread it. If there are issues with the information in the CRF, the CRA can fill out a query sheet and give it to the CRC and site PI. The CRC can then modify the data according to any revisions made by the site PI. After confirming that the database is correct, the site PI, CRC, and statistician will lock the database.

All documents that contain participants’ personal information will be identified by code number and stored separately. Only researchers involved in this study can be available to the confidential documents.

All study data, including the paper-based documents and electronic data, will be stored at each site for 5 years after the completion of the trial.

Because the study is not a drug trial and the sponsor or funder has no access to the raw data, a data monitoring committee will not be formed, and there is no planned trial audit. Besides, there are no interim analyses and stopping guidelines due to the very low risks of adverse events and other unintended effects.

### 2.15 Quality control

Quality control will be carried out throughout the trial to maintain quality. Monitoring information will be regularly submitted to the site PI and retained for future reference. The steering committee, composed of the 3 research centers’ PI, a statistician, and a foundation lead, is responsible for the coordination, development, and quality control of all the programs in the trial. All researchers must receive professional training on the trial method, study technique, and the method used for regular monitoring before participating in the trial. The therapist will be tested after training to ensure consistency of methods. The steering committee will discuss any modifications or corrections to the study protocol and submit them to the ethics committee for approval. Detailed records of changes will be kept.

### 2.16 Statistical analyses

The analysis set will consist of a full analysis set (FAS), a per-protocol set, and a safety set. The safety set will include randomly assigned participants and receive at least one intervention treatment. The FAS will consist of data indicating that the treatment was as close to the intention to treat as possible. In addition to meeting the safety set criteria, the participants must have been evaluated for the primary outcome at least once at the baseline. The FAS will be used in the primary analysis. For missing data, we will analyze the underlying reason and use an imputation adjustment approach, and the last observation carried forward analysis will be used to handle the missing data. After the primary analysis, a sensitivity analysis will be performed by comparing the results from the per-protocol analysis and the intention to treat analysis to evaluate the impact of missing data on the trial results. Besides, subgroup analyses by the center will be done.

(1) Statistical analysis method
  For all of the analyses, SAS version 9.4 was used. The confidence interval will be established at 95%, and the significance level at 0.05.
  Continuous variables were analyzed with an ANOVA test if normally distributed (results are presented as means ± SD) or Kruskal-Wallis H test if not normally distributed (presented as median with interquartile range). Multiple comparisons were addressed using the Tukey post hoc test (for ANOVA), and Bonferroni correction (for Kruskal–Wallis). The classification count data is checked by the chi-square test. This study used a one-sided test. When *P* < .05, the difference was considered statistically significant.
(2) Statistical analysis content
  a. Baseline evaluation: Social demographic characteristics, baseline severity analysis.
  b. Efficacy evaluation: We use the low back pain VAS score sheet, spinal ROM sheet, ODI scale, SF-36 scale, and the number of treatment sessions.
  c. Safety evaluation: Evaluation was made by the incidence of adverse events or recurrence of pain, or further injury from accidents during the follow-up period, and the level of safety.
  d. Compliance evaluation: Evaluation was carried out using the compliance rate.

## 3 Discussion

CNLBP is a significant health issue worldwide. Given its prevalence, the need to assess practical approaches for low back disorders is of prime importance. TN is one of the essential parts of TCM and has contributed to people's health in China for thousands of years. A considerable body of scientific evidence supports such therapies for CNLBP treatment, but their efficacy has not been established.^[35]^ Therefore, it is essential to provide robust evidence about the effectiveness of TN for CNLBP.

The present trial is a comparative effectiveness study of TN (intervention) and PT (control) for pain relief and functional recovery in patients with CNLBP. We evaluated 3 aspects of CNLBP: pain, physical function, and quality of life. The VAS will be used as the primary outcome. The secondary outcomes are the ODI, which measures dysfunction and includes 10 questions on daily activities; the ROM, which evaluates functional limitations in everyday life; and the SF-36, which measures changes in health-related quality of life.

According to the theory of TCM,^[35]^ good physical health depends on qi and blood circulation. The common causes of pain are stagnation of qi and stasis of blood. Moreover, other pathogenic factors such as phlegm and dampness can be identified as causative factors in a blockage. TN can relieve pain by promoting qi and blood's local and systemic circulation by removing pathogenic factors. Studies have shown that TN may alleviate CNLBP by reducing inflammation and repairing damaged mitochondria in the skeletal muscle.^[36]^

The results of this RCT will contribute to the clinical practice of TN for CNLBP. And the results will be presented in an open-access peer-reviewed journal and an academic conference.

## Acknowledgments

The authors thank the HEAD Foundation in Singapore for providing excellent facilities to undertake this study.

## Author contributions

**Data curation**: Xuan Zhou, Juan Yang, and Qing-yu Ma.

**Investigation**: Xuan Zhou, Juan Yang, Qing-yu Ma, Yu Guo, Cheong Kwok Chee Philip, Tay Boon Keng, Long-bin Shen, and Ke-jie He.

**Methodology**: Jia-xu Chen, Brent A. Bauer, Tan Ia Choo Celia.

**Resources**: Yu Guo, Cheong Kwok Chee Philip, Tay Boon Keng, Long-bin Shen, and Ke-jie He.

**Software operating:** Qiao Fan.

**Supervision**: Jia-xu Chen, Brent A. Bauer, Tan Ia Choo Celia.

**Writing** – **original draft**: Xuan Zhou, Juan Yang, and Qing-yu Ma.

**Writing** – **review & editing**: Jia-xu Chen, Brent A. Bauer, Tan Ia Choo Celia.

## Supplementary Material

Supplemental Digital Content
