# Supplementary material for: The effectiveness of Tuina in managing chronic non-specific low back pain: A protocol of a multicenter international randomized controlled trial
Source: Medicine (Baltimore). 2022 Feb 18;101(7):e28883. doi: 10.1097/MD.0000000000028883 (PMC9281981; doi:10.1097/MD.0000000000028883)
Supplement: Supplemental Digital Content [file medi-101-e28883-s001.docx]

Appendix 1 Intervention

Tuina group

In this study arm, the therapist will administer a nine-step protocol intended to ease LBP and release the low back's soft tissue. The specific protocol used is described below:

***Step 1: Dredging the meridians and collaterals***

Ask the patient to take a prone position. Standing on one side of the patient, apply rolling manipulation from the lumbar region to the lower limbs. Apply palm root-kneading manipulation on spinal erector muscles on both sides of the back and lumbosacral area. Continue kneading the lumbosacral and lower limb regions along the Bladder Meridian of Foot Taiyang. (10 minutes)

***Step 2: Pushing on the back and lumbosacral region***

Ask the patient to take a prone position. Standing on one side of the patient, apply palm-pushing manipulation along Governor Vessel (from DU14 (dàzhuī) to DU2 (yāoshù)) and bilateral courses of Bladder Meridian of Foot Taiyang (from BL11 (dàzhù) to BL30 (báihuánshù)) from the back to the lumbar region, for five times respectively. (2 minutes)

***Step 3: Plucking manipulation to relieve spasms***

Ask the patient to take a prone position. Standing on one side of the patient, apply plucking manipulation with the thumb (Extend the thumb straight, the tip of the thumb is taken as the working point, the other four fingers are placed on the relative areas to assist the thumb, press the thumb on the side of tendons or muscles, then make a transverse plucking movement, perpendicular to the tendons or muscular fibers, like plucking the string of a musical instrument) or plucking manipulation with the palm and thumb (Extend the thumb straight, the tip of the thumb is taken as the working point, the palm of the other hand is placed on the thumb (working point) to exert force, press the thumb on the side of tendons or muscles, then make a transverse plucking movement to the vertical tendons or muscular fibers, like plucking the string of a musical instrument) on sacrospinalis muscles of the back and lumbosacral region bilaterally. Repeat the manipulation up and down several times, using an amount of strength that the patient can accept. The degree of treatment is based on the patient's tolerance level. (4 minutes)

***Step 4: Pointing-pressing acupoints to relieve pain***

Ask the patient to take a prone position. Standing on one side of the patient, apply pointing-pressing manipulation on BL17 (géshù), BL23 (shènshù), BL25 (dàchángshù), BL27 (xiǎochángshù), ashi point, DU4 (mìngmén), DU3 (yāoyángguān), GB30 (huántiào), BL40 (wěizhōng), KI3 (tàixī), SI3 (Selection of acupoints based on syndrome differentiation) located on the back, lumbosacral and leg region till the patient feels soreness and distention. (6 minutes)

***Step 5: Pulling-stretching manipulation of the waist***

Ask the patient to take a prone position. And hold the top side of the bed with both hands forcefully, the tuina practitioner stands at the foot side of the bed to pull the patient's body by holding his ankles with both hands and gradually increasing force. The movements of pulling-stretching manipulation should be stable and gentle. The strength applied increases from mild to strong during pulling to avoid accidents. (1 minute)

***Step 6: Pulling manipulation to adjust joints***

The patient takes a lateral recumbent position, with the leg on the lower side extended and the upper leg bent at the hip and knee joint placed over the other leg. The Tuina practitioner places an elbow at the patient's shoulder and the other on the back of the hip on the same side. Twist the patient's waist by pushing the shoulder and pulling the hip in opposite directions until there is resistance. (2 minutes)

***Step 7: Rotating the waist to relax the lumbar muscles***

The patient takes a supine position with flexed knees and hips. Pressing two knees with one hand to close the knees together and holding the lower parts or ankles of the legs with the other hand, rotate the bent legs clockwise or counterclockwise for 3-5 times respectively to lead the movement of the waist. (1 minute)

***Step 8: Scrubbing manipulation to warm the back and lumbosacral region***

Ask the patient to take a prone position. Standing on one side of the patient, apply scrubbing manipulation using the palm root of the hand (vertical scrubbing manipulation) along with the bilateral courses of Bladder Meridian located on the back and lumbosacral region till a warm sensation is felt at the local area. Then apply scrubbing manipulation using the hypothenar region of the palm (transverse scrubbing manipulation) on the lumbosacral region until a warm sensation is felt at the local area. (3 minutes)

***Step 9: Patting the waist to end the treatment***

Ask the patient to take a prone position. Standing on one side of the patient, apply to pat manipulation on the lower back, lumbosacral, buttock and lower limb in a regular rhythm. (1 minute)

Physical therapy group

PT treatment for chronic low back pain will follow the following general protocol:

(1) A provisional functional diagnosis of the subject is made, which will enable the Physiotherapist to decide on a treatment plan specific to the subject's symptoms.

(2) A treatment plan based on the symptoms and functional impairments signs of the subject is developed through clinical reasoning and may include the following treatment options:

a. Manual therapy: Joint mobilization or manipulation may be used to reduce joint stiffness or pain from the low back.

i. Joint-specific mobilization versus soft tissue mobilization as needed.

ii. Lumbar manipulation as needed.

b. Electrophysical agents (EPA): Various electrophysical agents may be used to reduce pain, muscle tension and joint stiffness.

i. The types of EPA are Heat therapy (from various heating devices, e.g. hot packs, infra-red lamps etc.), ice (e.g. using ice packs, ice cubes, ice sprays etc.), ultrasound therapy, transcutaneous electrical sensory stimulation, lumbar traction (if needed) etc.

c. Education: Advice on posture and positions for functions at work, home, and sporting activities may be provided to help with ongoing therapy after treatment.

i. Posture/body mechanics – basic and simplified

ii. Stay active – it is safe to do so

iii. Limit sedentary time (bed rest)

iv. Manage stress, sleep well, and eat well to reduce pain

d. Aerobic exercise:

i. Graded exposure to aerobic exercise will follow the recommended American College of Sports Medicine (ASCM) guidelines of 20 to 30 minutes at any intensity that achieves 55 to 90% of the maximum heart rate, at least 3 days a week.

ii. Examples of aerobic exercise: Walking, swimming, bicycling, etc. per patient comfort/preference

iii. Advance exercise intensity to 30 to 60 minutes per session, 5-7 days per week.

These exercises will not be performed in all PT sessions. Subjects will be instructed to achieve this on their own as part of a home exercise program. PT sessions are limited in time to 30 mins per session. These exercises are recommended for the subjects and are not necessarily done during each PT session.

e. Flexibility and core strengthening exercises (see below): A customized series of exercises to provide stretching of soft tissues and strengthening of the core muscles of the spine and peripheral tissues will be taught to the subject.

i. Low back stretch and rotation stretch

ii. Abdominal bracing

iii. Bridge

iv. Quadruped arm/leg lifts

v. Plank

***Home Exercise Program and Advice***

Home exercises to maintain the effects of treatment will also be provided so that subject can continue their treatment at home. Exercise materials may be provided for the subject so that they can review the positions, duration and number of repetitions at home until the next therapy session.

Tuina combined Physiotherapy group

The patients receive TN treatment or PT treatment every session. TN or PT should not be performed in the same session. The sequence for the treatment does not matter.
